# Mesenteric Lymph Node Transplantation in Mice to Study Immune Responses of the Gastrointestinal Tract

**DOI:** 10.3389/fimmu.2021.689896

**Published:** 2021-07-26

**Authors:** Haroon Shaikh, Juan Gamboa Vargas, Zeinab Mokhtari, Katja J. Jarick, Maria Ulbrich, Josefina Peña Mosca, Estibaliz Arellano Viera, Caroline Graf, Duc-Dung Le, Katrin G. Heinze, Maike Büttner-Herold, Andreas Rosenwald, Joern Pezoldt, Jochen Huehn, Andreas Beilhack

**Affiliations:** ^1^Interdisciplinary Center for Clinical Research (IZKF) Experimental Stem Cell Transplantation Laboratory, Würzburg University Hospital, Würzburg, Germany; ^2^Department of Internal Medicine II, Würzburg University Hospital, Würzburg, Germany; ^3^Graduate School of Life Sciences, Würzburg University, Würzburg, Germany; ^4^Rudolf Virchow Center, Julius-Maximilians-University Würzburg, Würzburg, Germany; ^5^Department of Nephropathology, Friedrich-Alexander-Universität Erlangen-Nürnberg, Erlangen, Germany; ^6^Institute of Pathology, Julius-Maximilians-University Würzburg, Würzburg, Germany; ^7^Comprehensive Cancer Centre Mainfranken, Würzburg University Hospital, Würzburg, Germany; ^8^Laboratory of Systems Biology and Genetics, Institute of Bioengineering, School of Life Sciences, Ecole Polytechnique Fédérale de Lausanne (EPFL), Lausanne, Switzerland; ^9^Department of Experimental Immunology, Helmholtz Centre for Infection Research, Braunschweig, Germany; ^10^Cluster of Excellence RESIST (EXC 2155), Hannover Medical School, Hannover, Germany

**Keywords:** acute graft-versus host disease, alloreactive T cells, mesenteric lymph node, lymph node transplantation, mouse models, lymph node stromal cells

## Abstract

Mesenteric lymph nodes (mLNs) are sentinel sites of enteral immunosurveillance and immune homeostasis. Immune cells from the gastrointestinal tract (GIT) are constantly recruited to the mLNs in steady-state and under inflammatory conditions resulting in the induction of tolerance and immune cells activation, respectively. Surgical dissection and transplantation of lymph nodes (LN) is a technique that has supported seminal work to study LN function and is useful to investigate resident stromal and endothelial cell biology and their cellular interactions in experimental disease models. Here, we provide a detailed protocol of syngeneic mLN transplantation and report assays to analyze effective mLN engraftment in congenic recipients. Transplanted mLNs allow to study T cell activation and proliferation in preclinical mouse models. Donor mLNs proved viable and functional after surgical transplantation and regenerated blood and lymphatic vessels. Immune cells from the host completely colonized the transplanted mLNs within 7-8 weeks after the surgical intervention. After allogeneic hematopoietic cell transplantation (allo-HCT), adoptively transferred allogeneic CD4^+^ T cells from FVB/N (H-2^q^) mice homed to the transplanted mLNs in C57BL/6 (H-2^b^) recipients during the initiation phase of acute graft-versus-host disease (aGvHD). These CD4^+^ T cells retained full proliferative capacity and upregulated effector and gut homing molecules comparable to those in mLNs from unmanipulated wild-type recipients. Wild type mLNs transplanted into MHCII deficient syngeneic hosts sufficed to activate alloreactive T cells upon allogeneic hematopoietic cell transplantation, even in the absence of MHCII^+^ CD11c^+^ myeloid cells. These data support that orthotopically transplanted mLNs maintain physiological functions after transplantation. The technique of LN transplantation can be applied to study migratory and resident cell compartment interactions in mLNs as well as immune reactions from and to the gut under inflammatory and non-inflammatory conditions.

## Introduction

Lymph nodes (LNs), spleen and Peyer’s patches (PPs) are secondary lymphoid organs that serve as sites for immune cell interaction and activation. LNs are unique in morphology and function. These filter-like structures continuously scan lymph content that moves from the afferent lymph into the subcapsular lymph node sinus and serve as spatially highly organized hubs of immune cell interactions (1). After recognition of a pathogenic antigen (Ag), an immune response is mounted, whereas in case of dietary or commensal antigens, tolerance is induced.

Surgical LN transplantation is a pre-clinical tool that has been instrumental to study tissue-resident LN stromal cells (LNSCs) in the immune microenvironment of LNs as well as their role in the modulation of cells of hematopoietic lineage under homeostatic and inflammatory conditions (2–5). In seminal work, others have utilized LN transplantation to demonstrate that LNSCs regulate peripheral tolerance (6) and that stromal cells of mLNs imprint gut-homing properties on antigen-responsive T cells to express α4β7 integrin and CC chemokine receptor 9 (CCR9) (5, 7). Furthermore, mLN stroma shapes resident dendritic cells (DCs) to attain high Treg-inducing capacity soon after birth in a Bmp2-dependent manner (8).

Worbs and colleagues elegantly showed oral tolerance induction exclusively takes place in the mLNs with antigens transported from the intestinal surface by DCs through the afferent lymphatics, whereas roles of PPs in the induction of oral tolerance were dispensable (9). Oral tolerance is in part mediated by the generation of FoxP3^+^ Tregs (peripherally induced Tregs, pTregs) converted from conventional FoxP3^-^ CD4^+^ T cells (10–14).

Notably, mLNs not only serve as sites for oral tolerance to food antigens but form an important firewall blocking systemic dissemination of microbes as potential pathogens and priming of intestinal immune cells by mounting effective immune responses (15, 16). mLNs drain lymph from various sites of GIT including the small intestine (SI) and colon, however different nodes of mLNs are anatomically segregated of lymphatic drainage from the SI and colon (17–19).

As pointed out above, lymphocytes primed in mLNs are imprinted for gut tropism and accordingly the ligands specific for their homing receptors α4β7 integrin and CCR9 are found in the gastrointestinal tract (GIT) (5, 7, 20, 21). These allow for efficient homing of immune effector cells to the lamina propria of the intestinal tract *via* the blood stream to protect from intestinal infections but are also relevant in pathological inflammatory conditions such as inflammatory bowel disease (17, 22–27) and intestinal acute graft-versus-host disease (aGvHD) (28–36). Therefore, we performed surgical mLNs transplantation to study the mucosal immune system of the gut in physiology and disease conditions.

Here, we provide an in-depth methodological description of the surgical mLNs transplantation procedure in C57BL/6 mice. We show that donor mLNs are viable after surgical transplantation, retain their histologic immune architecture, topological organization, normal vascular and lymphatic function. Furthermore, by transplanting mLNs from B6.CD45.1 congenic donor mice (B6.CD45.1) revealed the kinetics of repopulation of transplanted mLNs with all lineages of host-type hematopoietic cells. Transplanted mLNs provided all the required stimuli for the effective proliferation, differentiation and expansion of CD4^+^ T cells similar to non-transplanted mLNs, e.g., in a mouse model of aGvHD even in otherwise MHCII deficient hosts.

Hence, the described procedure is a suitable technique to study complex and dynamic immune cell interactions within the alimentary tract.

## Material and Equipment

### Materials

Materials are listed in **Table 1** corresponding to the experimental procedures.

**Table 1:** Materials used for the LN transplantation and associated techniques.

Materials	Catalog #	Company
**Mesenteric lymph node transplantation operation**		
Anesthetic solution		
1 ml syringe 26GA x 3/8’’ (0.45 mm x 10 mm), BD Plastipak™	300015	Becton Dickinson
1 ml insulin syringe 30GA x 1/2’’ (0.3 mm x 12 mm), Omnican^®^ 100	9151141	Braun
Fibrin sealant (TISSEEL, Fibrin Sealant kit with DUPLOJECT system)	1504516	Baxter
Eye ointment (Bepanthen^®^)		Bayer
Analgetic (Carprofen)	53716-49-7	Midas Pharma GmbH
Hair removal cream		Müller
Sterile razors	704028	Body products, Relax Pharma u. Kosmetik GmbH
Sterile dissecting swab (Setpack^®^ size 2)	12780	Lohmann & Rauscher
Sterile gauze swab (Gazin^®^ 5 cm x 5 cm)	13695	Lohmann & Rauscher
Sterile cotton swab (Rotilabo^®^)	EH12.1	Carl Roth
Operation towel	800430	BARRIER, Mölnlycke Health Care
Pasteur pipettes	2600111	Neolab
70% ethanol	T931.3	Carl Roth
Quickpad^®^ 70% 2-propanol		Holtsch Medizinprodukte GmbH
Povidone iodine (Bruanol^®^ 7.5% solution)	3864065	Braun
0.9% NaCl (Aqua ad iniectabilia)	14NM32	Fresenius Kabi Deutschland
Suture, 6-0 with beveled needle	V301G	Ethicon
**Cell isolation**		
DPBS without Ca^2+^/Mg^2+^	P04-36500	Pan Biotech
Sterile scalpel blades, feather # 10	BB510	B. Braun
Disposable serological pipettes	760180, 607180, 606180	Greiner Bio-one
Pipette controller (Accu-jet^®^ pro)	26300	Brandt
Cell counting chamber (Neubauer)	ZK03	Hartenstein
Micropipettes	042760930, 642752433, 942741768, 342733754, 042720454, 942711302	VWR
Cell strainer, 70 μm EASYstrainer™	542070	Greiner Bio-one
Tube 50 ml	227261	Greiner Bio-one
Microtubes 1.5 ml	72.706	Sarstedt
Erythrocyte lysis buffer		
DNase I	10104159001	Roche
Collagenase VIII	C2139	Sigma Aldrich
Collagenase P	11213865001	Roche
Dispase II	4942078001	Roche
Trypan blue solution	T8154-100ML	Sigma Aldrich
Magnetic cell enrichment buffer		
**Antibodies**		
Anti-CD3ε antibody (145-2C11) coupled to APC	100312	Biolegend
Anti-CD3 antibody (SP7)	RBG024	Zytomed
Anti-CD4 antibody (RM4-5) coupled to PE	100512	Biolegend
Anti-CD4 antibody (RM4-5) coupled to PerCP/Cy5.5	100540	Biolegend
Anti-CD8a antibody (53-6.7) coupled to PerCP/Cy5.5	100734	Biolegend
Anti-CD11c antibody (N418) coupled to AF647	117312	Biolegend
Anti-CD19 antibody (6D5) coupled to APC/Cy7	115530	Biolegend
Anti-CD19 antibody (D4V4B)	90176S	Cell Signaling
Anti-CD24 antibody (M1/69) coupled to PerCP/Cy5.5	101824	Biolegend
Anti-CD25 antibody (PC61) coupled to APC	102012	Biolegend
Anti-CD31 antibody (MEC13.3) coupled to AF488	102514	Biolegend
Anti-CD31 antibody **(**390**)** coupled to biotin	102404	Biolegend
Anti-CD31 antibody (SZ31)	DIA 310	Dianova
Anti-CD44 antibody (IM7) coupled to PE	103008	Biolegend
Anti-CD45 antibody (30-F11) coupled to PerCP/Cy5.5	103132	Biolegend
Anti-CD45.1 antibody (A20) coupled to APC-Cy7	110716	Biolegend
Anti-CD45.2 antibody **(**104**)** coupled to AF488	109816	Biolegend
Anti-CD62L (L-Selectin) antibody (MEL-14) coupled to PE-Cy7	25-0621-82	Invitrogen
Anti-CD90.1 antibody (OX-7) coupled to APC-Cy7	202519	Biolegend
Anti-LPAM-1 (Integrin α4β7) antibody (DATK32) coupled to PE	120606	Biolegend
Anti-MAdCAM-1 antibody (MECA-367) coupled to biotin	120706	Biolegend
Anti-Podoplanin antibody (8.1.1) coupled to APC	127410	Biolegend
Anti-Ter119 antibody (M1/69) coupled to PerCP/Cy5.5	116228	Biolegend
Anti-Ki67 antibody (16A8) coupled to AF647	652408	Biolegend
**Commercial kits**		
LIVE/DEAD™ Fixable Violet Dead Cell Stain Kit	L34955	Thermo Fisher
Foxp3/Transcription Factor Staining Buffer Set	00-5523-00	eBioscience
CD4^+^ T cell enrichment kit (Dynabeads™ Untouched™ Mouse CD4 Cells Kit)	11415D	Thermo Fisher
T cell depletion kit (CD90.1 MicroBeads, mouse and rat)	130-121-273	Miltenyi Biotec
Streptavidin, Alexa Fluor™ 750 conjugate	S21385	Invitrogen
Streptavidin, Alexa Fluor™ 546 conjugate	S11225	Invitrogen
**Other consumables**		
V-bottom 96-well plate		
Normal rat serum	10710C	Thermo Fisher
Evans blue	E2129	Sigma-Aldrich
D-Lucifirin, potassium salt	7903	BioVision
Diphtheria toxin from *Corynebacterium diphtheriae*	D0564-1MG	Sigma-Aldrich
**Light sheet fluorescence microscopy**		
Fetal bovine serum (FBS)	10099, 10100	Gibco
Paraformaldehyde, granulated	0335.3	Carl Roth
Triton® X 100	3051.4	Carl Roth
*n*-hexane	139386-500 ml	Sigma Aldrich
Benzyl benzoate	B6630-1 l	Sigma Aldrich
**Histology**		
Target Retrieval Solution, Citrate pH 6.1 (10x)	S1699	Dako Agilent
VECTASTAIN^®^ Elite ABC-HRP Kit, Peroxidase (Standard)	PK-6100	Vector laboratories
ImmPACT^®^ DAB	SK-4105	Vector laboratories
Mayer´s hemalum for counter-staining	109249	Merck
**Equipment**		
ISMATEC Reglo analog pump	ISM795C	IDEX Health and Science LLC
Stereo microscope	SZ51	Olympus
Light source	KL1500 LCD	Schott
Isis animal shaver	GT420	Braun
X-ray irradiation source	CP-160	Faxitron
Surgery tool: 2 fine forceps, 1 pair of small scissors, 1 flexible needle holder		Karl Hammacher GmbH and mergo
2 heating mats (20 cm x 30 cm)	76085	Trixie Heimtierbedarf GmbH
Infrared lamp	BF 27	Beurer
Thermometer (dual thermo max/min)	E609790	Amarell Electronic
Centrifuge (Megafuge 40R)		Thermo Scientific
Water bath	WNB 14	Memmert
Attune NxT flow cytometer equipped with 405, 488, 561 and 638 nm lasers and an autosampler		Thermo Scientific
IVIS Spectrum	124262	Perkin-Elmer
Gas anesthesia system for IVIS imaging platform	XGI-8	Perkin-Elmer
Light sheet fluorescent microscope (LSFM)	Home build setup	(37, 38)
**Software**		
FlowJo	Version X	TreeStar
Imaris	Versions 7.7.2 and 8.1.1	Bitplane
Living Image^®^	Version 4.0	Perkin-Elmer
Matlab	Version R2016a	Mathworks
**Mice**		
C57BL/6 (C57BL/6NCrl)	Strain code 027	Charles River
FVB/NCrl (FVB)	Strain code 207	Charles River
FVB.L2G85		Bred in-house
C57BL/6.*Tyrc-2J* (B6 albino)		Bred in-house
C57BL/6.L2G85.CD45.1		Bred in-house
C57BL/6.L2G85.DsRed		Bred in-house
C57BL/6.129S2-*H2^dlAb1-Ea^*/J	Stock no. 003584	Bred in-house
C57BL/6.Tg(Itgax-DTR/OVA/EGFP)1Garbi	MGI no. 463655	Bred in-house

### Preparation of Reagents

- Anesthetic: add 2 ml of Xylavet^®^ (20 mg/ml, CP-Pharma) and 2 ml of Ursotamin^®^ (100 mg/ml, Serumwerk) to 21 ml of DPBS. PromAce^®^ (10 mg/ml, Boehringer Ingelheim) was prepared separately and used for experiments involving surgery. Inject 10 μl/g of body weight to reach desired concentration. (Xylazin 16 mg/kg, Ketamine 80 mg/kg and Acepromazine 2 mg/kg).
- Analgesic: Carprofen (5 mg/kg) to relieve pain 30 min before opening the abdominal cavity and, if needed, every 12 h up to third day after the surgery.
- Erythrocyte lysis buffer: dissolve 89.9 g of NH_4_Cl, 10 g of KHCO_3_, and 0.37 g EDTA in 1 l of autoclaved, deionized water.
- Trypan blue solution: dissolve 1 g of trypan blue in 100 ml of PBS. Dilute 1:10 in PBS to get working solution to mix at equal volume with cell suspension.
- Magnetic cell enrichment buffer: 0.375 g of EDTA and 0.5 g of BSA in 500 of DPBS, sterile-filter.
- FACS buffer: 0.375 g of EDTA and 5 ml of FBS in 500 ml of DPBS.
- FACS blocking buffer: 20% normal rat serum in DPBS.
- Luciferin: 5 g D-lucifirin in 165 ml of 0.9% NaCl (Aqua ad iniectabilia), stored at -20°C.
- 4% PFA solution: 4 g PFA in 100 ml DPBS, dissolved at 65°C, pH: 7.4
- LSFM clearing solution (BABB): 1:2 ratio of benzyl alcohol and benzyl benzoate.
- Enzyme mix for lymph node digestion: RPMI-1640, 0.8 mg/ml Dispase II, 0.3 mg/ml Collagenase P and 0.15 mg/ml DNase I.
- Enzyme mix for small intestine digestion: 10 ml solution (1.5 mg/ml Type VIII Collagenase dissolved with 40 μg/ml of DNase I in pre-warmed HBSS Ca^2+^/Mg^2+^, 2% FCS.

## Stepwise Procedures

This protocol focuses on syngeneic mLN transplantation from a C57BL/6 mouse into another C57BL/6 mouse and the subsequent analysis of successful engraftment and the functionality of the transplanted mLN to initiate an immune response in aGvHD inflammatory settings. Therefore, these sections are in greater detail, whereas additional procedures are outlined in brief.

Animal experiments were performed according to project license number 55.2.2-2532.2-1038-9, which was approved by the Regierung von Unterfranken, Würzburg.

### Mesenteric Lymph Node Transplantation

We performed mLN transplantation in 8- to 12-week-old C57BL/6 mice from syngeneic donors expressing different reporter transgenes (firefly luciferase or fluorescent proteins) or congenic markers. Preference was given to males over female animals, as they recovered faster from the operation.

### Preparation

Donor and recipient were anesthetized with an intraperitoneal (i.p.) injected mixture of Ketamine (80 mg/kg body weight) and Xylazine (16 mg/kg body weight), one day prior to the operation. To allow for sterile conditions at the site of surgery, ventral fur was wetted with 70% ethanol using a dissecting swab, trimmed with a shaver and removed with hair removal cream. Alternatively, mice were anesthetized with 2% isoflurane in O_2_ (XGI-8 gas anesthesia system, Perkin-Elmer) during hair removal.

On the day of operation, two sterile working places were prepared: one for the donor mLN isolation and recipient preparation while the other one for the mLN transplantation operation. For each of the spaces, a heating mat was pre-warmed and covered with a sterile OP towel, all needed surgical instruments and reagents were sterilized and laid out.

An operation cover was prepared by cutting an OP towel to a size of 15 cm x 15 cm, and an oval window was cut into the center with a diameter of 1.5 cm x 2.5 cm to be placed on the operation site.

A gauze pad was prepared by folding it to 7.5 cm x 5 cm and incising a 2 cm long central slit.

A clean recovery cage was placed under an infrared lamp, and the sensor of thermometer was placed on the bedding to monitor the local temperature and not let it rise above 30°C to avoid overheating.

### Operation and Lymph Node Transplantation

Mice were anesthetized with an i.p. injected mixture of Ketamine (80 mg/kg body weight), Xylazine (16 mg/kg body weight) and Acepromazine (2 mg/kg body weight) (39) dissolved in PBS (200 – 250 μl) and placed on a sterile, heated OP towel for preparation. The animals were subcutaneously (s.c.) injected with the perioperative analgesic Carprofen (5 mg/kg) to relieve pain 30 min before opening the abdominal cavity and every 12 h up to the third day after the surgery if required. Eyes were protected from dehydration by applying eye ointment - Bepanthen^®^ - Augen- und Nasensalbe (Bayer Vital GmbH, Leverkusen, Deutschland). The shaved abdomen was wetted with 70% ethanol using a dissection swab; the skin was disinfected with Braunol^®^ (B. Braun, Melsungen, Germany) in an outward circling motion.

After 10 min, anesthetic depth was ensured to be stadium III.2 (surgical tolerance) by pinching the hind paw (plantar reflex). The dosage of the anesthetic used is usually very well tolerated and leads to a reliable depth of anesthesia up to stage III.2.

If in exceptional cases by checking the plantar reflex, anesthesia depth up to stage III.2 was not achieved, an i.p. additional dose of the anesthetic was provided (20% of the original dose). The volumes can easily be applied accurately using an insulin syringe. For a more precise dosage of the amount of anesthesia, the anesthetic was additionally diluted 1:1 in PBS so that the injection volume doubled.

**Figure 1:**
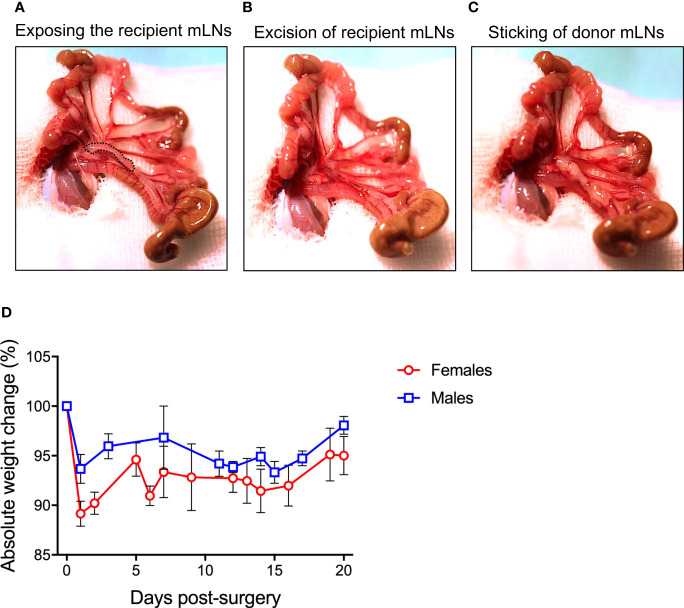
Procedure of mesenteric lymph node (mLN) transplantation. **(A)** Peritoneal cavity of the mice was carefully opened and the bowel along with the caecum taken out and laid on DPBS-wetted gauze pad to make all the mLNs lobes visible. **(B)** All the lobes of recipient mLNs were excised out avoiding bleeding from the superior mesenteric artery lying behind the mLNs **(C)** 10 μl of fibronectin were pipetted on the scar, donor mLNs soaked in thrombin were carefully placed on the scar and incubated for 3-5 min before internalizing the complete bowel for subsequent suturing. **(D)** Absolute weight change of male and female mice undergoing surgical intervention.

The prepared animal was transferred onto a fresh sterile and heated OP towel and the prepared OP-cover was applied. A 1-1.5 cm midline incision was made in the skin and in the peritoneum on the *linea alba*, no bleeding should be visible. The wound was covered with the prepared gauze pad, the slit overlaid on the incision. The pad was soaked with PBS to prevent drying of externalized intestinal tissue. Next, two cotton swabs were soaked with PBS and used to handle the intestinal contents. The swabs were inserted into the peritoneum to localize and gently exteriorize the caecum, which is located on the animals’ right side to the craniolateral incision. The intestinal tissue was handled with great care to prevent postoperative ileus. First, the caecum was placed on the gauze pad left to the incision, and the intestinal tissue was kept moist at all times by dripping PBS on it using a Pasteur pipette. The large intestine was then gently pulled out, starting from the caecum, until the mLN appeared attached to mesentery. The most distal part of small intestine was also gently pulled out to have visible access to the chain of mLNs and to identify the bottom node next to the caecum **(** **Figure 1A** **)**.

With the help of fine surgical scissors, the mLNs of the recipient mice were excised from the bottom to the top with minimal fat and connective tissue **(** **Figure 1B** **)**. Extra care was taken to avoid injuring the superior mesenteric artery lying behind the mLNs. Minor bleeding was stopped by holding the Setpack dissecting swabs (Lohmann & Rauscher, Rengsdorf, Germany) to wounded areas. The donor LN was joined to the mesenteric tissue using a two-component fibrin glue (TISSEEL (Baxter, Höchstadt, Germany): 10 μl of fibronectin (component to TISSEEL) was pipetted on the area from where the recipient mLN was excised. The donor mLNs (isolated with the same procedure as recipient mice) were dipped in thrombin solution (component to fibrin glue) and placed on the wound in the same bottom-to-top-orientation as removed recipient LN: from distal (from the caecum) to the proximal region (towards the ileum) **(** **Figure 1C** **)**. For firm adhesion, the glue was allowed to polymerize for 3-5 min. Subsequently, the small and large intestine along with the caecum were carefully placed back into the peritoneum. The repositioning in the correct orientation of intestinal loops was important to prevent a postoperative ileus. The peritoneum was closed with three non-consecutive stitches with coated VICRYL^®^ (polyglactin 910) suture (Ethicon, Dülmen, Germany). Subsequently, the skin was closed with four stitches. Bepanthen^®^ Wund- und Heilsalbe (Bayer Vital GmbH, 51368 Leverkusen, Deutschland) was applied on the closed surgical wound. The overall operation procedure took 15-25 min per animal. The mice were placed into a clean cage and held warm with an infrared lamp until recovered from anesthesia. The drinking water was supplemented with Baytril (Enrofloxacin, 0.05%) for 7 days after surgery to avoid infections.

### Lymphatic Drainage Assay

Mice were anesthetized with an i.p. injected mixture of Ketamine (80 mg/kg body weight) and Xylazine (16 mg/kg body weight) and Acepromazine (2 mg/kg) (39) dissolved in PBS (200 – 250 μl) and placed on a sterile, heated OP towel for preparation. The animals were s.c. injected with the perioperative analgesic Carprofen (5 mg/kg) to relieve pain 30 min before opening the abdominal cavity. To evaluate the lymphatic drainage from the bowel into the transplanted mLNs, 5 μl Evans blue dye (2% in DPBS) was injected into PPs of the ileum using 33-gauge needle with a Hamilton syringe under a SZ51 stereo microscope (Olympus, Hamburg, Germany) by gently holding the intestine with blunt forceps. Five min after injection, mLNs were photographed for Evans blue drainage.

### Preparation of Lymph Nodes for Light Sheet Fluorescent Microscopy (LSFM)

Mice were anesthetized with an i.p. injected mixture of Ketamine (80 mg/kg body weight) and Xylazine (16 mg/kg body weight) and Acepromazine (2 mg/kg) (39) dissolved in PBS (200 – 250 μl) and placed on a sterile, heated OP towel for preparation. The animals were s.c. injected of the perioperative analgesic Carprofen (5 mg/kg) to relieve pain 30 min before opening the abdominal cavity. For blood vessels staining, mice were retro-orbitally injected with 20 μg of anti-CD31 biotin antibody in 100 μl of DPBS, after 20 min mice received 10 μg of Streptavidin, Alexa Fluor™ 750 conjugate in 100 μl of DPBS.

After 20 min, anesthetic depth was ensured to be stadium III.2 (surgical tolerance) by pinching the hind paw. If anesthesia was insufficient, further anesthetic was provided retro-orbitally. Not more than 20-50 μl were injected at a time to avoid over-dosage. Prior to mLN extraction, mice were perfused for 2 min with DPBS and 8 min with paraformaldehyde (PFA) 4%. mLNs were further fixed for 2 hours in 4% PFA and washed with DPBS three times with 30 min incubations before processing at 4°C. The mLNs were blocked overnight in DPBS containing 2% FBS, 0.2% Triton X100. To stain for high endothelial venules (HEVs): Anti-MAdCAM-1 antibody (MECA-367) coupled to biotin at a dilution of 1:100 was used. Samples were incubated for 24 hours with gentle shaking at 4°C. On the next day, samples were washed with DPBS three times with 30 min incubations at 4°C and then incubated with Streptavidin, Alexa Fluor 546™ conjugate for 24 hours with gentle shaking at 4°C. After staining, mLNs were washed with DPBS three times with 30 min incubations at 4°C and dehydrated in a graded ethanol series (30%, 50%, 70%, 80%, 90%) for 90 min each at room temperature and in 100% overnight at 4°C. The following day, the samples were rinsed for 2 hours in 100% *n*-hexane; subsequently n-hexane was replaced stepwise by LSFM clearing solution. Special care was taken at this step to strictly avoid air exposure of the samples. mLN samples became optically transparent and suitable for LSFM imaging after incubation in the LSFM clearing solution for at least 2 hours at room temperature (30).

### LSFM Setup and Data Acquisition

The LSFM modular setup is home-built providing four excitation lines of 491, 532, 642 and 730 nm as described elsewhere (37). Images acquired by LSFM were analyzed on IMARIS software v8.1.1 (Bitplane AG, CA, USA). When required, background subtraction was applied in accordance with the diameter of the cell population to eliminate unspecific background signals.

### Allogeneic Hematopoietic Cell Transplantation

#### Donor T Cell Enrichment

Naïve T cells were enriched from the spleen of 8- to 12-week-old FVB.L2G85 (H-2^q^) donor mice (28, 40), expressing firefly luciferase and congenic cell markers (CD90.1 and CD45.1). Splenocytes were enriched for T cells with (Dynabeads™ Untouched™ Mouse CD4 Cells Kit, Thermo Fisher) according to the manufacturer’s protocols, counted by trypan blue exclusion, stained with Cell trace CFSE according to manufacturer protocol. Typically, T cell yields ranged between 15 to 30% of total splenocyte input with a final T cell purity of 90 to 97%.

#### Donor Bone Marrow Isolation and T Cell Depletion

Bone marrow cells were isolated from the hind limbs (femur and tibia) of 8- to 12-week-old wildtype donor FVB/NCrl mice. Cell numbers were determined by trypan blue exclusion and depleted of T cells by utilizing Thy 1.1 enrichment kit (Thermo Fisher), following manufacturer’s protocol. Typically, bone marrow cell yields ranged between 1 – 1.5 x 10^8^ cell per donor mouse, while T cell depletion purity was between 95 to 98%.

### Allogeneic Hematopoietic Cell Transplantation

Untreated C57BL/6 or C57BL/6 mice transplanted with mLNs were myeloablatively irradiated (9 Gy), and 1.2 x 10^6^ FVB.L2G85 donor T cells were intravenously injected *via* the retro-orbital venous plexus together with 5 x 10^6^ T cell-depleted bone marrow cells in a total volume of 200 μl. The drinking water was supplemented with Baytril (Enrofloxacin, 0.05%) for 7 days after transplantation to avoid infections. aGvHD was scored clinically and body weight was assessed daily.

### Diphtheria Toxin Mediated Cell Depletion

For DCs depletion in B6a.CD11c.DOG donor mice prior to the isolation of mLNs, animals were injected intraperitoneally (i.p.) with diphtheria toxin (Sigma-Aldrich, Hamburg, Germany) at doses of 20 ng/g body weight on day -5, -3, and -1 prior to the day of surgery.

### Histology

Formalin-fixed, paraffin-embedded (FFPE) specimens were processed for hematoxylin and eosin staining (H&E) as previously described (41) and immunohistochemical assessment of mLN architecture. Briefly, 1 µm paraffin sections were deparaffinized and rehydrated in graded ethanol. Antigen retrieval was performed in a steam cooker (Biocarta Europe, Hamburg, Germany) at 120°C for 2.5 min using a commercially available cooking buffer: target retrieval solution, citrate pH 6.1 (Catalog #, S1699, Dako Agilent). Primary antibodies, anti-CD3 (1:200, rabbit, monoclonal, clone SP7, Catalog # RBG024, Zytomed), anti-CD19 (1:500, rabbit, monoclonal, Catalog # 90176S, Cell Signaling) and anti-CD31 (1:200, rat IgG2a, monoclonal, Catalog # 15219/01, Dianova), were added over night at room temperature. After washing biotinylated secondary antibodies were incubated for 30 minutes at room temperature (all 1:500). For detection ECTASTAIN^®^ Elite ABC-HRP kit, peroxidase (standard), catalog # PK-6100 and ImmPACT^®^ DAB substrate, Catalog # SK-4105, (both Vector Laboratories) were used and Mayer´s hemalum for counter-staining (Catalog # 109249, Merck). Images were taken with Zeiss Axio imager A1 at 100x original magnification.

### Bioluminescence Imaging

*In vivo* bioluminescence imaging was performed with an IVIS Spectrum CCD-imaging system (Perkin-Elmer). Mice were anesthetized by injecting 10 μl of anesthetic solution (Ketamine and Xylazine) per gram of body weight intraperitoneally. D-Luciferin was injected in a concentration of 150 μg/g body weight and images were taken 10 min after the injection which allowed the identification of T cell proliferation and migration.

To perform *ex vivo* imaging mice were injected with the same mixture of anesthetic and D-Luciferin. 10 min after injection mice were euthanized and organs were removed within 4 min. *Ex vivo* images provided higher resolution of selective organ signal distribution. Bioluminescence imaging data was analyzed on Living image^®^ 4.5.5 software.

### Isolation of Cells

Day 3- and 6- after allo-HCT mice were sacrificed, and the mLNs, and small intestine were harvested for analysis.

### Cell Isolation From Lymph Nodes

Cells from the LNs were isolated using a protocol modified from Fletcher and colleagues (42). mLNs were pierced with a syringe needle 2 – 3 times and directly transferred into a centrifuge tube containing 2 ml RPMI-1640 medium on ice for further processing.

The RPMI-1640 medium was replaced with 2 ml freshly prepared enzyme mix containing 0.8 mg/ml Dispase II, 0.3 mg/ml Collagenase P and 0.15 mg/ml DNase I in RPMI-1640 medium.

Tubes were incubated at 37°C in a water bath for 20 min and gently inverted several times at 5 min intervals. To carefully disrupt the capsule after the first incubation step, the cell suspension was gently resuspended with a 1 ml pipette. Tissue pieces were allowed to settle down for 1 min and subsequently released leukocytes in the cell suspension were transferred into a new tube containing 10 ml FACS-buffer stored on ice.

2 ml of fresh enzyme-mix were added to the remaining mLNs pieces and mixed with a 1 ml pipette and again incubated for 10 min in a water bath at 37°C. After incubation, the cell suspension was again gently resuspended and released cells were transferred to the tube containing the previous supernatant in ice-cold FACS-buffer. For the last time, 2 ml of digestion-solution were added to the residual tissue fragments and this time vigorously mixed every 5 min using a 1 ml pipette until all fragments were digested. Collected supernatants, that have been stored on ice, were centrifuged at 400 g, 4°C for 5 min and counted.

### Cell Isolation From Small Intestine

T cells from the small intestine were isolated as described before (43). Small intestinal tissue from the stomach to caecum was excised. Fat and mesenteric tissue was cleared from the intestine, Peyer’s patches were removed, and intestine was washed with PBS. After removal of all fecal material, intestine pieces were cut into 2 cm pieces and transferred to a 50 ml centrifuge tube containing 20 ml medium 2 - HBSS medium (Ca-/Mg-, 2 mM EDTA, 5% FCS) and incubated for 20 min at 37°C with gentle rotation (100 RPM).

After incubation, gut pieces were passed through a 70 µm cell strainer over a new 50 ml centrifuge tube. The flow-through, containing the intraepithelial cells were stored on ice. Remaining gut pieces were transferred back into the tube containing medium 2 and again incubated for 20 min in a thermal incubator at 37°C with gentle rotation (100 RPM). This step disrupted the remaining intraepithelial fraction form the underlying mucosa. After incubation, gut pieces were again passed through a 70 µm cell strainer and pooled with the first fraction. This cell fraction contained the gut intraepithelial lymphocytes (IELs). IELs fraction was centrifuged at 400 g for 5 min at 4°C, resuspended in appropriate volume and counted.

To further process *lamina propria*, the remaining intestine pieces on the cell strainer were transferred onto a plastic weighing pan and reduced to pulp using fine dissection scissors. The gut-pulp was transferred to a new 50 ml centrifuge tube containing 10 ml of digestion solution: 1.5 mg/ml Type VIII Collagenase (Sigma, Hamburg, Germany) dissolved with 40 μg/ml of DNase I (Sigma, Hamburg, Germany) in pre-warmed HBSS Ca^2+^/Mg^2+^, 2% FCS) and incubated at 37°C for 20 min under vigorous rotation (200 RPM). Following incubation, cell suspension was vortexed for 30 secs and passed through a 100 µm cell strainer (Miltenyi Biotec, Gladbach, Germany). The cell strainer was rinsed with 30 ml of ice-cold Ca^2+^/Mg^2+^ HBSS containing 10% FCS as washing-buffer and centrifuged at 500g for 10 min at 20°C. Cell pellet was resuspended in 10 ml PBS for counting.

### Flow Cytometry

Up to 1 x 10^6^ cells were stained per well in 96-well v-bottom plates. Cells were resuspended in 100 μl of blocking solution and incubated for 5 min at 4°C. 100 μl of antibody mix were added and cells were stained for 30 min at 4°C in the dark. Cells were pelleted at 400 g for 5 min 4°C and resuspended with FACS buffer for acquisition on the flow cytometer.

For intracellular staining cells were fixed, permeabilized and stained with Foxp3/Transcription Factor Staining buffer set (eBioscience) following the manufacturer’s protocol.

### Statistical Analysis

Data are shown as mean ± standard deviation (SD). Different groups were compared by two-tailed unpaired student’s t-tests using GraphPad Prism 8 software (La Jolla, CA, USA). Level of significance was set at p < 0.05.

## Results

### Donor mLNs Sustain Functionality After Surgical Transplantation

**Figure 2:**
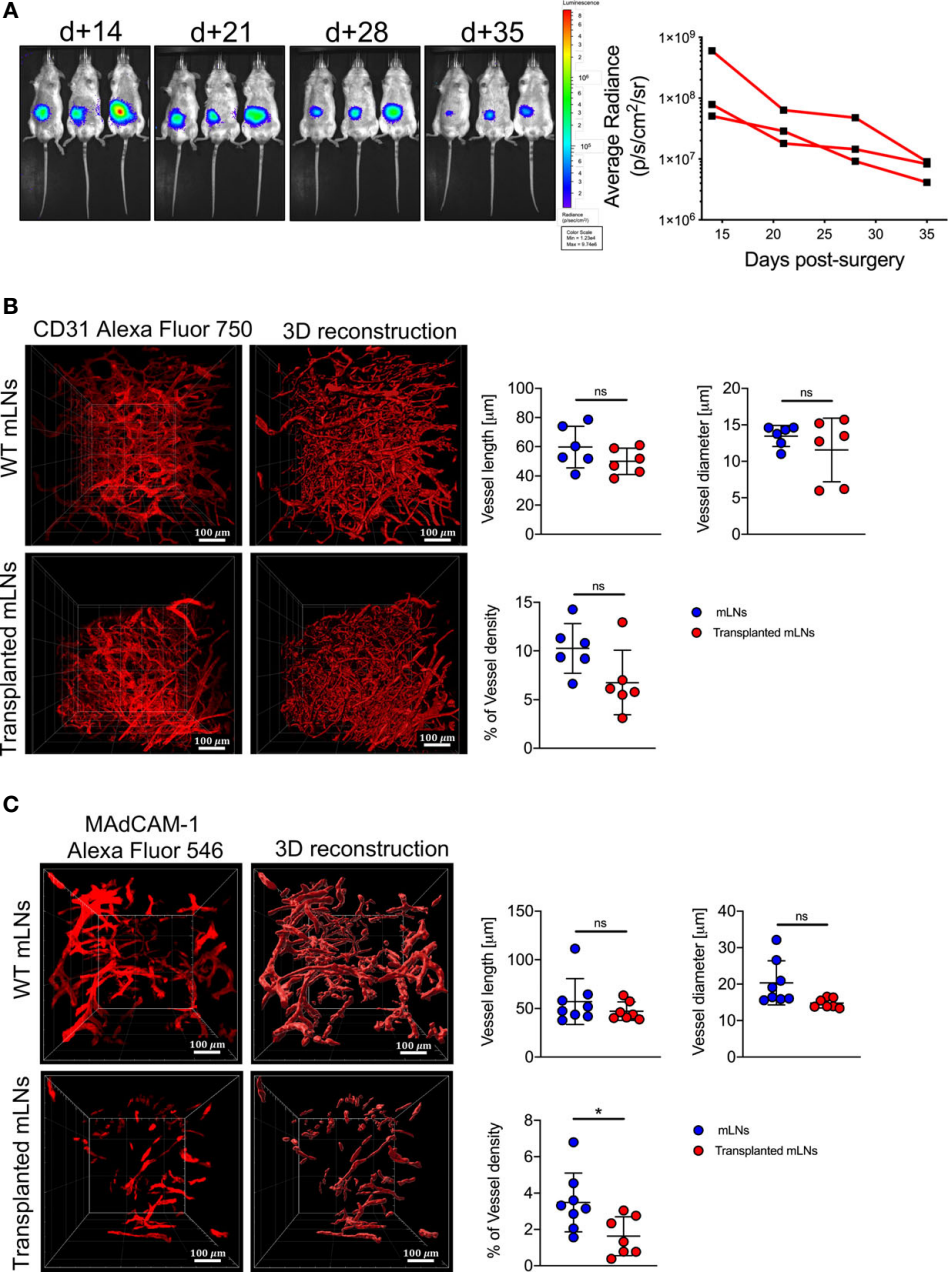
Donor mLNs engrafted in the recipient mice after surgical transplantation. **(A)** Bioluminescence signals emitted from luc^+^ mLNs from B6.L2G85 mice transplanted into a B6 albino recipient mouse and quantification (p/s/cm^2^/sr) of BLI signal from donor luc^+^ mLNs. **(B)** Zoomed LSFM images of blood vessels staining with anti-CD31 antibody and 3D reconstruction (20x objective, scale bar = 100 μm) and quantification of blood vessel length, diameter and density were analyzed from reconstructed images on Imaris, 5 weeks after mLN transplantation. **(C)** Zoomed LSFM images of HEVs staining with anti-MAdCAM-1 antibody and 3D reconstruction (20x objective, scale bar = 100 μm) and quantification of HEVs vessel length, diameter and density were analyzed from reconstructed images on Imaris, 5 weeks after mLN transplantation. Each data point represents one mouse, data pooled from two experiments; unpaired non-parametric Mann-Whitney test, (Mean± SD); *p < 0.05; ns, not significant.

Mice undergoing surgical transplantation of mLNs lost approximately 10% of body weight post-surgery, however they recovered 5% body weight within five days (**Figure 1D**). First, we determined the viability of mLNs after surgical transplantation, in this set of experiments mLNs from B6.L2G85.CD45.1 expressing firefly luciferase were transplanted into congenic B6 albino mice. The bioluminescence signal from the transplanted cells was initially measured at day 14 post-surgery and sustained signal was acquired even until 5 weeks (35 days) post-surgery (**Figure 2A**). The decrease in bioluminescence signal can be explained by the efflux of hematopoietic cells from the LN *via* vascular and lymphatic system subsequent to successful engraftment.

To assess the functionality of the donor mLN and its connection with the vascular system of the mouse, we employed LSFM and stained the blood vessels with CD31. The vascularization of blood vessels in the transplanted mLNs as measured by the length, diameter and density was comparable to the mLNs from wild-type mice **(** **Figure 2B** **)**, suggesting adequate neovascularization and adjoining of small blood vessels to the mesenteric artery after surgery. To determine if the transplanted mLNs retained a functional post-capillary venous system, we performed 3D-LSFM imaging of mucosal vascular addressin cell adhesion molecule 1 (MAdCAM-1), which revealed HEVs length and diameter was not altered in the surgically transplanted mLNs however density of HEVs in the transplanted LNs was significantly reduced **(** **Figure 2C** **)**. In conclusion, these findings reveal that the transplanted mLNs retain physiological vascular function after surgical transplantation.

### Transplanted mLNs Retain Normal Morphology and Drain Lymph From the PPs

**Figure 3:**
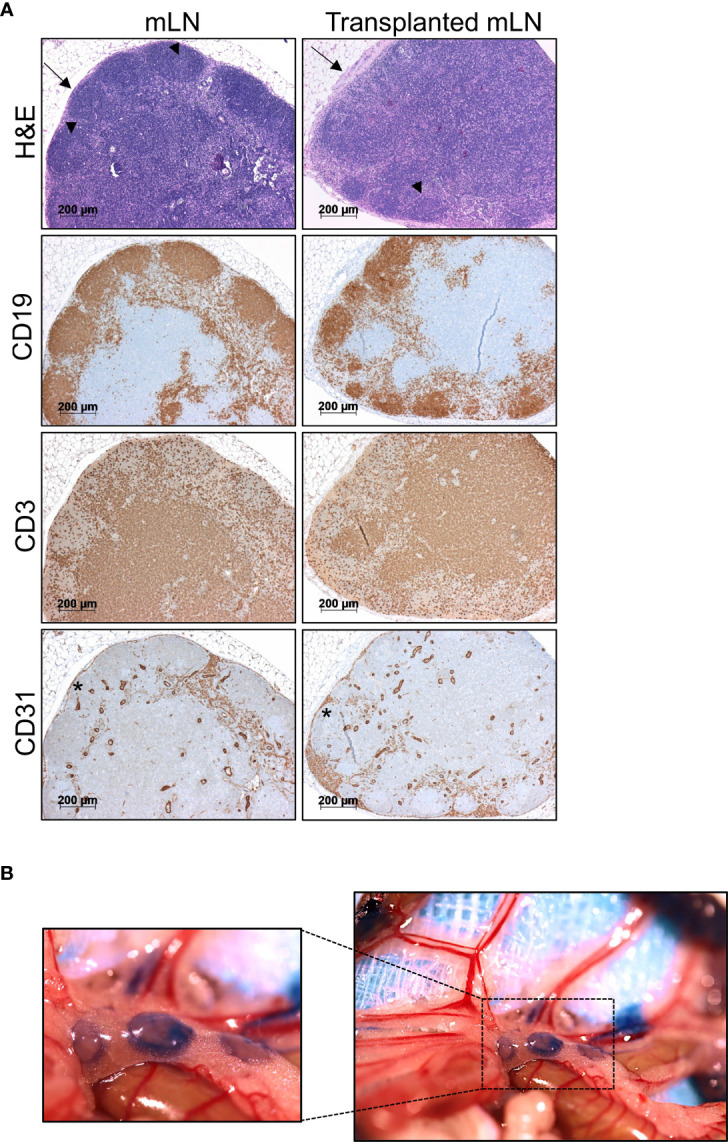
Transplanted mLNs retain normal morphology, cell distribution and lymphatics. **(A)** H&E stainings of transplanted vs. non-transplanted mLNs reveal a regular immune architecture with B cell follicles and some germinal centers (arrowheads) in both settings in the cortex and T cell zones (top). Lymph node capsules (arrow) display signs of capsule fibrosis in transplanted mLNs. Immunohistochemical analysis of CD19^+^ B cells confirm proper B cell follicles in the cortex and distribution of CD3^+^ T cells dominating in the paracortical areas. CD31 staining on LECs in the subcapsular sinus (asterisk) revealed an intact structure of the mLN capsule, 5-6 weeks after mLN transplantation. **(B)** Donor mLNs lymph vessels conjoin to recipient’s intestinal lymphatic vasculature after transplantation. Evans blue injected into the iliac Peyer’s patches drains into the transplanted mLNs, 5 weeks after mLN transplantation.

To evaluate the morphology and immune architecture of the transplanted mLNs, we performed H&E stainings. We observed that the lymph node architecture was preserved in transplanted mLNs similar to non-transplanted mLNs: CD19^+^ B cells distributed regularly and accentuated in follicles in the cortex whereas CD3^+^ T-cells predominantly located in the paracortical area. In both, transplanted and non-transplanted mLNs displayed some germinal centers (**Figure 3A**). CD31 staining revealed no obvious differences in the vasculature, moreover CD31 staining on LECs in the subcapsular sinus revealed intact structure of the mLN capsule, which is necessary for the lymphatic drainage from afferent lymphatics to the medullary sinuses. As the only remarkable difference we observed signs of fibrosis of the mLN capsule in transplanted animals as opposed to untreated animals (**Figure 3A**).

To test, whether functional lymphatic anastomoses formed in transplanted mLNs, we evaluated lymphatic drainage from the small intestines. To visualize lymphatic vessels and to assess efficient lymphatic drainage we injected Evan’s blue into the jejunal and iliac Peyer’s patches, which drained to the transplanted mLNs **(** **Figure 3B** **)**. Staining of lymph vessels and immediate drainage revealed Evans blue labeled to mLNs indicating that functional lymphatic vessel anastomoses had formed with transplanted lymph nodes.

### Donor mLNs Are Populated by Recipient Hematopoietic Cells After Transplantation

**Figure 4:**
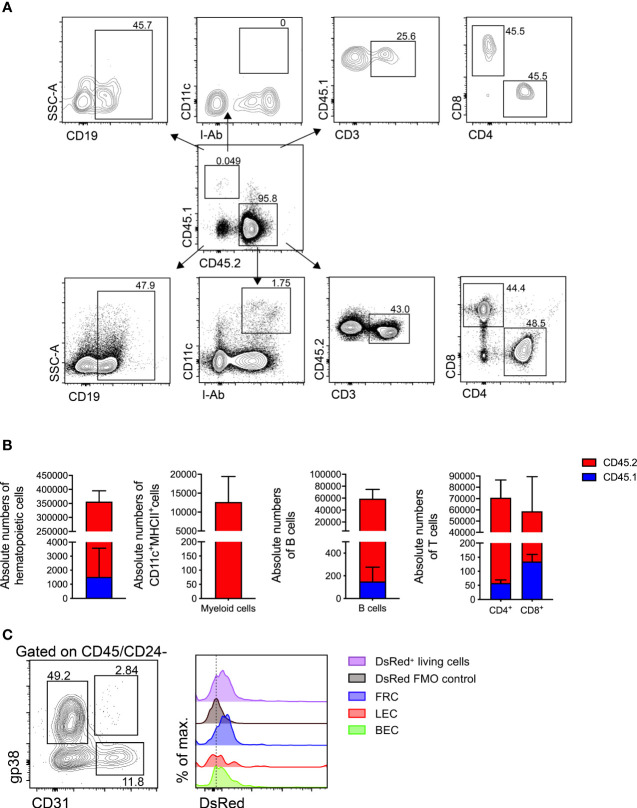
Donor mLNs are repopulated with recipient hematopoietic cells after surgical transplantation. **(A)** Flow cytometry gating of hematopoietic cells in mLNs from B6.CD45.1 mouse transplanted in a B6.CD45.2 mouse. **(B)** Absolute numbers of hematopoietic cells, myeloid cell (dendritic cells and macrophages), B cells and CD3^+^ pre-gated CD4^+^ and CD8^+^ T cells in CD45.1^+^ and CD45.2^+^ sub-populations, 5 weeks after mLN transplantation. Data pooled from five mice analyzed in two experiments. **(C)** Flow cytometry gating of non-hematopoietic LNSCs and DsRed fluorescence signal from B6.DsRed mLNs transplanted in a B6.WT mouse, with DsRed^+^ living cells from a DsRed^+^ mouse as positive and living cells from a WT mouse serving as negative controls. Analysis performed 5 weeks after mLN transplantation. Error bars represent Mean± SD.

To address whether donor mLNs would be colonized by donor hematopoietic cells or by recipient hematopoietic cells after surgical transplantation, we transplanted mLNs from B6.L2G85.CD45.1 into congenic B6.WT (CD45.2) mice. Five weeks post-surgery the animals were euthanized and analyzed for the CD45.1^+^ and CD45.2^+^ population in mLNs by flow cytometry **(** **Figure 4A** **)**. The vast majority of hematopoietic cells in the transplanted mLNs (95.4%) were of host (CD45.2) origin when compared to 0.47% of donor (CD45.1) origin. We could not detect CD45.1^+^ myeloid cells (CD11c^+^MHCII^+^), however we detected very few remaining CD45.1^+^ T cells (CD3^+^CD4^+^ and CD3^+^CD8^+^) in the transplanted mLNs **(** **Figure 4B** **)**. The proportion of host hematopoietic cells found in the transplanted mLNs was relatively similar to the frequency in untreated C57BL/6 mice **(** **Figure S1** **)**.

To further probe the origin of non-hematopoietic LN stromal and endothelial cells we employed transgenic mice that ubiquitously expressed a red fluorescent reporter gene and transplanted mLNs from these B6.DsRed donor mice into a B6.WT recipients. After successful engraftment we could confirm fibroblastic reticular cells (FRCs), lymph endothelial cells (LECs) and blood endothelial cells (BECs) **(** **Figure S2A** **)** based on their emission of DsRed protein fluorescence supporting that they originated from the donor mLNs **(** **Figure 4C** **).**

In summary, transplanted mLNs were entirely repopulated with recipient hematopoietic cells while retaining LNSCs of donor origin within five weeks of transplantation.

### Transplanted mLNs Sustain Normal T Cell Activation and Proliferation

**Figure 5:**
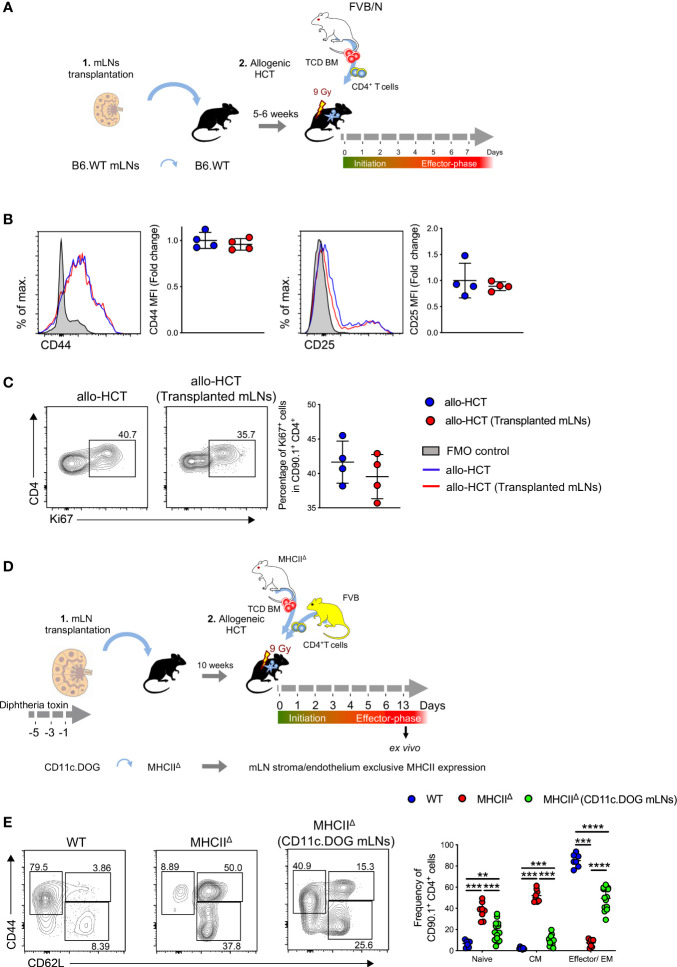
Alloreactive CD4^+^ T cells are robustly activated in the transplanted mLNs under aGvHD inflammatory conditions. **(A)** Experimental strategy: mLNs from B6.WT mice were transplanted into B6.WT recipient; 5-6 weeks post-surgery; MHC major mismatch aGvHD was induced by lethal irradiation (9 Gy) and allogenically transplanting with 5x10^6^ TCD BM cells and 5x10^6^ CD4^+^ T cells from FVB/N mice. **(B)** Mean fluorescence intensity (MFI) of CD44 and CD25 on donor T cells d+3 of allo-HCT. **(C)** Proliferative capacity of donor CD4^+^ T cells were analyzed by expression of Ki67 d+3 of allo-HCT. **(D)** Experimental strategy: mLNs of B6.MHCII^Δ^ mouse were surgically removed and transplanted with donor mLNs from B6.CD11c.DOG mice that had been depleted of CD11c^+^ cells by i.p. administration with diphtheria toxin 20 ng/gram body weight day -5, -3, and -1 day before surgery. 10 weeks post-surgery MHCII^Δ^ (CD11c.DOG mLNs), untreated B6.WT and MHCII^Δ^ mice were myeloablatively irradiated with 9 Gy and i.v. transplanted with 5x10^6^ T cell-depleted (TCD) BM and 5x10^6^ CD4^+^ T cells from MHCII^Δ^ and FVB mice respectively. **(E)** Analysis for T cell subsets - CD44 and CD62L (pre-gated on CD4^+^CD90.1^+^) on day +13 of allo-HCT from mLNs. Each data point represents one mouse, data pooled from two experiments; unpaired non-parametric Mann-Whitney test, (Mean± SD); **p < 0.01, ***p < 0.001 and ****p < 0.0001.

To assess whether transplanted mLNs are fully functional and can initiate an adaptive immune response, we employed a murine model of aGvHD. After allo-HCT, we evaluated alloreactive T cell activation and proliferation in mice with and without previously transplanted mLNs **(** **Figure 5A** **)**. In the initiation phase of aGvHD (day 3 of allo-HCT) (28), alloreactive donor CD4^+^ T cells as gated in **(** **Figure S2B** **)** expanded in the transplanted mLNs and upregulated the expression of T cell activation markers CD44 and CD25 **(** **Figure 5B** **)**, the donor CD4^+^ T cells in the transplanted mLNs upregulated Ki67 protein, indicating active proliferation comparable to WT group **(** **Figure 5C** **)**.

Day 6 of allo-HCT is considered as an effector phase of aGvHD, at a time-point at which the alloreactive T cells already infiltrate the aGvHD target organs (28). At this time point we detected similar BLI signal intensities derived from luciferase^+^ donor T cells and absolute number of alloreactive CD4^+^ T cells **(** **Figure S3A** **)**, indicating extensive alloreactive CD4^+^ T cell expansion in transplanted mLNs **(** **Figure S3B** **)** and other aGvHD target organs, similar to allo-HCT control recipients without previous mLN transplantation. Furthermore, donor CD4^+^ T cells upregulated T cell activation maker CD44 and intestinal homing receptor integrin α4β7 **(** **Figure S3C** **)** required for the T cell migration into the intestine and functional proliferation as measured by the upregulation of Ki67 **(** **Figure S3D** **)**. Moreover, allogenic CD4^+^ T cells differentiated into effector, effector memory and central memory T cells based on differential expression of CD44 and CD62L (L-Selectin) **(** **Figure S3E** **)**. At the same time-point we evaluated the activation status of allogeneic donor CD4^+^ T cell infiltrating the small intestines, an organ whose immune response is especially regulated by mLNs. Likewise, here we could not detect any differences in the allogenic CD4^+^ T cell activation and expansion that might be a result due to surgically transplanted mLNs **(** **Figures S4A–C** **)**.

Next, we asked whether non-hematopoietic cells of the mLNs can prime allogenic CD4^+^ T cells in the absence of professional hematopoietic antigen presenting cells (APCs). To this end we isolated mLNs from CD11c.DOG mice that had been depleted of CD11c^+^ cells by the administration of diphtheria toxin (DTx) **(** **Figure S5** **)** and surgically transplanted these mLNs into a complete MHCII deficient animal (MHCII^Δ^). Subsequently we transplanted allogeneic MHCII^Δ^ TCD BM and FVB CD4^+^ T cells into these MHCII^Δ^ mice harboring transplanted MHC competent CD11c.DOG mLNs as well as MHCII^Δ^ recipients without transplanted mLNs **(** **Figure 5D** **)**. In MHCII^Δ^ mice harboring transplanted MHC competent mLNs we observed priming of allogeneic donor CD4^+^ T cells that differentiated into effector/effector memory T cells (CD44^high^CD62L^-^). In contrast, allogeneic donor CD4^+^ T cells mostly displayed a naïve (CD44^-^CD62L^high^) and central memory (CD44^high^CD62L^high^) phenotype on day +13 of allo-HCT **(** **Figure 5E** **)**.

Taken together these experiments reveal the transplanted mLNs were able to induce an effective T cell response under inflammatory conditions, even in MHCII deficient hosts.

## Discussion

Strategically positioned between the layers of the mesentery, mLNs locate in the center of the GIT and their dysfunction (44) or lymphadenectomy (45, 46) disrupts gut immunity. In rats, it has been demonstrated that mLNs excision protects from lethal GvHD (47, 48). Others have shown that LN transplantation can decrease pathology of lymphedema (49–51).

Here we detailed a protocol of mLN transplantation and show that this surgical procedure is a feasible method to study the immune system of the gut in steady-state or under inflammatory conditions. Transplanted mLNs are engrafted into the host mesenteric tissue within a few weeks after surgery, achieving a functional mesenteric vascular and lymphatic system.

We surgically removed all mLNs from healthy C57BL/6 mice and replaced them with mLNs from different donor mice depending on the experimental question. It is to be pointed out that here we used healthy mice as recipients, nevertheless mice already under experimental intervention can be used as recipients. However, care has to be taken as the surgical procedure is quite intensive and might result in animal mortality during or after the surgical procedure. Extended troubleshooting guideline are displayed in **Table 2**.

**Table 2:** Troubleshooting guide.

Step	Problem	Possible reason	Solution
Anesthesia	Mice reacts to footpad pinching	Anesthesia underdosed	Slowly add small doses of anesthetic i.p. until desired anesthetic depth is achieved
	Mice take longer than 1 h to recover from anesthesia	Anesthesia overdose	Do not use more anesthesia than needed to reach desired anesthetic depth
		Low body temperature	Monitor the body temperature with a rectal probe. Place the animal in its cage in front of infrared lamp (but avoid overheating)
Lethality up to 24 hr after operation despite recovery from anesthesia	Post-operative ileus	Rough handling of the bowel	Handle bowels gently and with care. Always use pre-wetted cotton swabs to handle the bowels. Refrain from pinching the bowl with forceps. Do not apply pressure to the bowel. Keep bowel lubricated at all times
	Intestinal bleeding	Aneurysm at the site of mLN removal	Care should be practiced not to rupture/cut blood vessels while removing the host mLNs. If bleeding occurs, quickly stop the bleeding by holding Setpack**^®^** size 2 at the site of bleeding until coagulation occurs. Inject 200 – 300 μl solution of 0.9% NaCl s.c. to maintain blood volume
	Peritonitis	Introduction of infection during the operation procedure	Work aseptically according to (52)
		Introduction of infection after wound closure	Suture both peritoneum and the skin sufficiently
Donor mLNs placement into recipient mesenteric tissue	Intestinal loops stuck together with fibrin glue	Excessive use of fibrin glue	Pipette minimal amount of fibronectin at the site of removed mLNs

The transplanted mLNs in the recipient mice were viable after surgical procedure, which we confirmed with non-invasive bioluminescence imaging detecting the emission of signals from luciferase-transgenic donor mLNs. The transplanted mLNs retained physiological vascular and lymphatic function, suggesting the adjoining of blood vessels of the donor mLNs to the mesenteric artery and connection of the transplanted mLNs lymphatics vessels to the lymphatic system of the intestine within weeks after surgery. This was consistent with the report on axillary LN transplantation by Aschen and colleagues (49). However, we observed a reduction in HEVs density in transplanted mLNs when compared to endogenous non-transplanted mLNs. This could be attributed to a certain degree of vessel atrophy, as in our described procedure vessels from the host were not surgically conjoined to the donors rather, they joined spontaneously after resorption of fibrin glue during the engraftment phase. Furthermore, the transplanted mLNs maintained normal morphology with intact B cell follicles and T cell zones. However, we observed fibrosis on the capsule of transplanted mLNs, which could be explained by the deposition of fibrin glue during the surgical mLN transplantation procedure. Importantly, transplanted mLN retained their full capacity of lymphatic drainage from the intestinal tract. In line with our observations, others have shown that LN transplantation induces lymphangiogenesis, however lymphatic vessels induced by LN transplantation are abnormal in appearance but are functional and are able to transport lymph fluid and also cells (53).

Within a few weeks after surgical transplantation, the host hematopoietic cells of different lineages populated the transplanted mLNs, the efflux of donor derived hematopoietic cells resulted in the decrease of bioluminescence signal emitted by luc^+^ donor cells over the course of observation. The residual bioluminescence signal that we acquired stemmed obviously from the tissue resident stromal and endothelial cells of the transplanted mLNs. Hammerschmidt and colleagues also demonstrated that donor non-hematopoietic stromal and endothelial cells survive the surgical transplantation and that donor- and host-derived stromal cells were unevenly distributed in the transplanted LNs (7).

Whether to maintain peripheral tolerance under steady-state conditions or to mount an effective immune response under inflammatory conditions, different immune cells interact and are activated in a highly regulated microenvironment of secondary lymphoid organs. To assess the functional capacity of transplanted mLNs to induce an efficient adaptive immune cell response, we employed a murine allo-HCT model that resulted in allogeneic T cell activation and their migration to the target tissues in aGvHD. Donor CD4^+^ T cells effectively migrated from the blood to the transplanted mLNs in the initiation phase of aGvHD (day 3) and were activated and proliferated comparably to that in the mLNs from untreated animals. Furthermore, we observed similar CD4^+^ T cell activation and upregulation of gut homing receptors on CD4^+^ T cells in the transplanted mLNs indicating robust T cell priming, which was ensued by effector T cell infiltration in the *lamina propria* of the small intestine without time delay in the effector phase of aGvHD (day 6) (28–30). Taken together, these findings suggest that orthotopic syngeneic mLNs transplantation did not affect subsequent allogenic T cell activation under inflammatory conditions of aGvHD.

In recent years, a crucial role of non-hematopoietic APCs in aGvHD has emerged as it has been shown that DCs or B cells are dispensable in the initiation of aGvHD and allogenic activation of CD4^+^ T cells (54–56). Moreover, it was recently proposed that intestinal epithelial cells have the capacity to provide allo-antigen to allogenic CD4^+^ T cells (57). Here, we transplanted mLNs depleted of CD11c^+^ APCs into MHCII^Δ^ mice and observed allogenic CD4^+^ T cells priming within these mLNs. Notably, our data suggest that non-hematopoietic LNSCs in mLNs can provide priming signals to allogenic CD4^+^ T cells under aGvHD like inflammatory conditions. However, it still remains to be determined, which subtype of LNSCs can initiate alloreactive CD4^+^ T cell responses.

In contrast to previous LN transplantation studies on axillary LNs (49), popliteal LNs (8) and inguinal LNs (58), the use of fibrin glue served as a biological adhesive in our study and supported the engraftment of donor mLNs into the mesenteric tissue. Different nodes of mLNs drain from the jejunum, ileum and the colon (17–19) but in this study we did not elucidated if the orthotopically transplanted mLNs retained the similar lymph drainage pattern of distinct intestinal segments after surgical transplantation.

In conclusion, here we provide an elaborate protocol along with the necessary assays to perform successful mLNs transplantation in mice. This protocol can be used to study the role of resident LN stromal cells of mLNs in homeostasis (8, 12) and inflammatory conditions (6) and their interaction with the hematopoietic cells of the innate and adaptive immune system. Furthermore, mLNs being a major site of gut mucosal immunity regulation, this protocol provides an alternate tool to study gut immunology. With minor adjustments, this technique can also be applied to different disease models in mice to study e.g., the impact of microbiota and metabolites on particular stromal LN environments, modification of migrating antigen-presenting cells by distinct LN microenvironments or in cancer metastasis.

## Data Availability Statement

The raw data supporting the conclusions of this article will be made available by the authors, without undue reservation.

## Ethics Statement

The animal study was reviewed and approved by Regierung von Unterfranken.

## Author Contributions

HS designed, planned and carried out the experiments, analyzed and interpreted the data, and wrote the manuscript. ZM analyzed and interpreted the LSFM data. KJ assisted with establishing the surgery model. JV, MU, JM, EV, CG, and D-DL helped with the experiments. AR and MB-H performed histopathologic tissue analyses. EV and KH supported LSFM imaging. JP and JH provided intellectual support. AB designed the study, interpreted the data, reviewed and co-wrote the manuscript. All authors contributed to the article and approved the submitted version. 

## Funding

This work was supported by grants from the German research council (DFG) to AB (TRR221, 324392634; GRK2157 P1, 270563345) and Bayerische Forschungsstifung (Fortither WP2TP3) and the Europäische Founds für Regionale Entwicklung (EFRE; Center for personalized molecular immunotherapy). The Helmholtz Institute for RNA-based Infection Research (HIRI) supported this work with a seed grant through funds from the Bavarian Ministry of Economic Affairs and Media, Energy and Technology (Grant allocation # 0703/68674/5/2017 and 0703/89374/3/2017).

## Conflict of Interest

The authors declare that the research was conducted in the absence of any commercial or financial relationships that could be construed as a potential conflict of interest.

The reviewer MB has declared a shared affiliation with one of the authors JH to the handling editor at the time of review.

## Publisher’s Note

All claims expressed in this article are solely those of the authors and do not necessarily represent those of their affiliated organizations, or those of the publisher, the editors and the reviewers. Any product that may be evaluated in this article, or claim that may be made by its manufacturer, is not guaranteed or endorsed by the publisher.
