# Supplementary material for: Mesenteric Lymph Node Transplantation in Mice to Study Immune Responses of the Gastrointestinal Tract
Source: Front Immunol. 2021 Jul 26;12:689896. doi: 10.3389/fimmu.2021.689896 (PMC8352558; doi:10.3389/fimmu.2021.689896)
Supplement: Supplementary file 1 [file Presentation_1.pptx]

## Slide 1
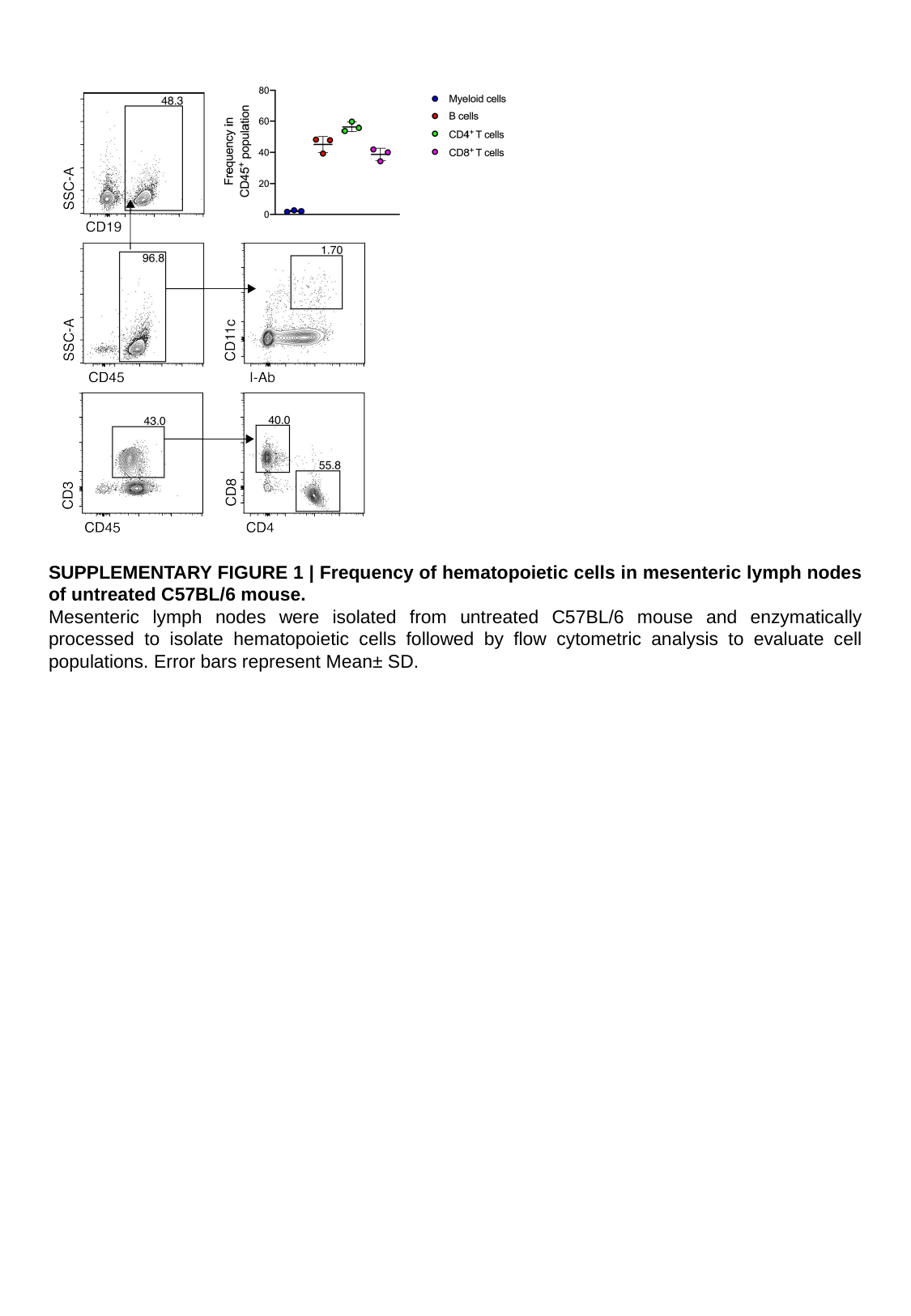

SUPPLEMENTARY FIGURE 1 | Frequency of hematopoietic cells in mesenteric lymph nodes of untreated C57BL/6 mouse.
Mesenteric lymph nodes were isolated from untreated C57BL/6 mouse and enzymatically processed to isolate hematopoietic cells followed by flow cytometric analysis to evaluate cell populations. Error bars represent Mean± SD.

## Slide 2
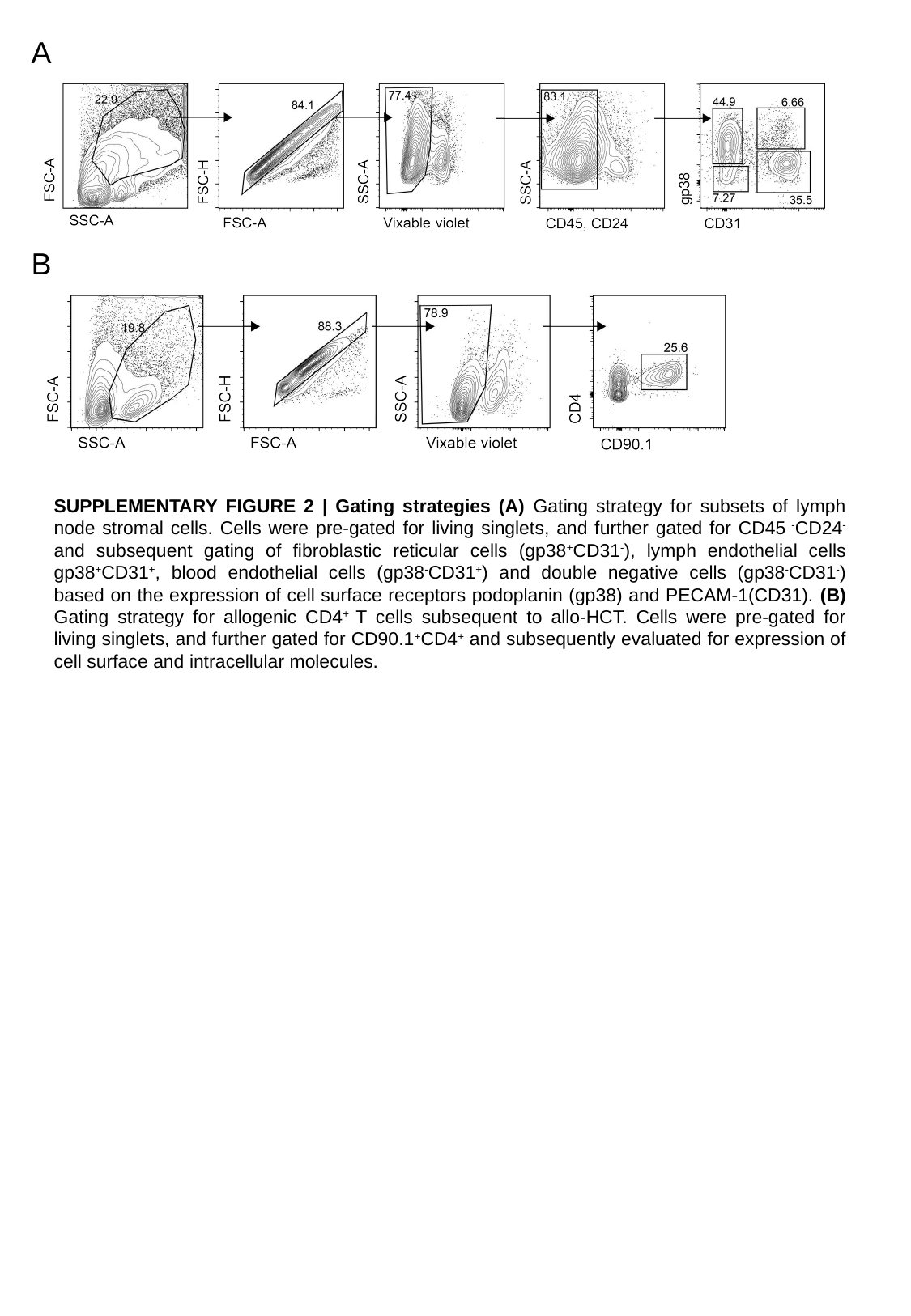

A
B
SUPPLEMENTARY FIGURE 2 | Gating strategies (A) Gating strategy for subsets of lymph node stromal cells. Cells were pre-gated for living singlets, and further gated for CD45 -CD24- and subsequent gating of fibroblastic reticular cells (gp38+CD31-), lymph endothelial cells gp38+CD31+, blood endothelial cells (gp38-CD31+) and double negative cells (gp38-CD31-) based on the expression of cell surface receptors podoplanin (gp38) and PECAM-1(CD31). (B) Gating strategy for allogenic CD4+ T cells subsequent to allo-HCT. Cells were pre-gated for living singlets, and further gated for CD90.1+CD4+ and subsequently evaluated for expression of cell surface and intracellular molecules.

## Slide 3
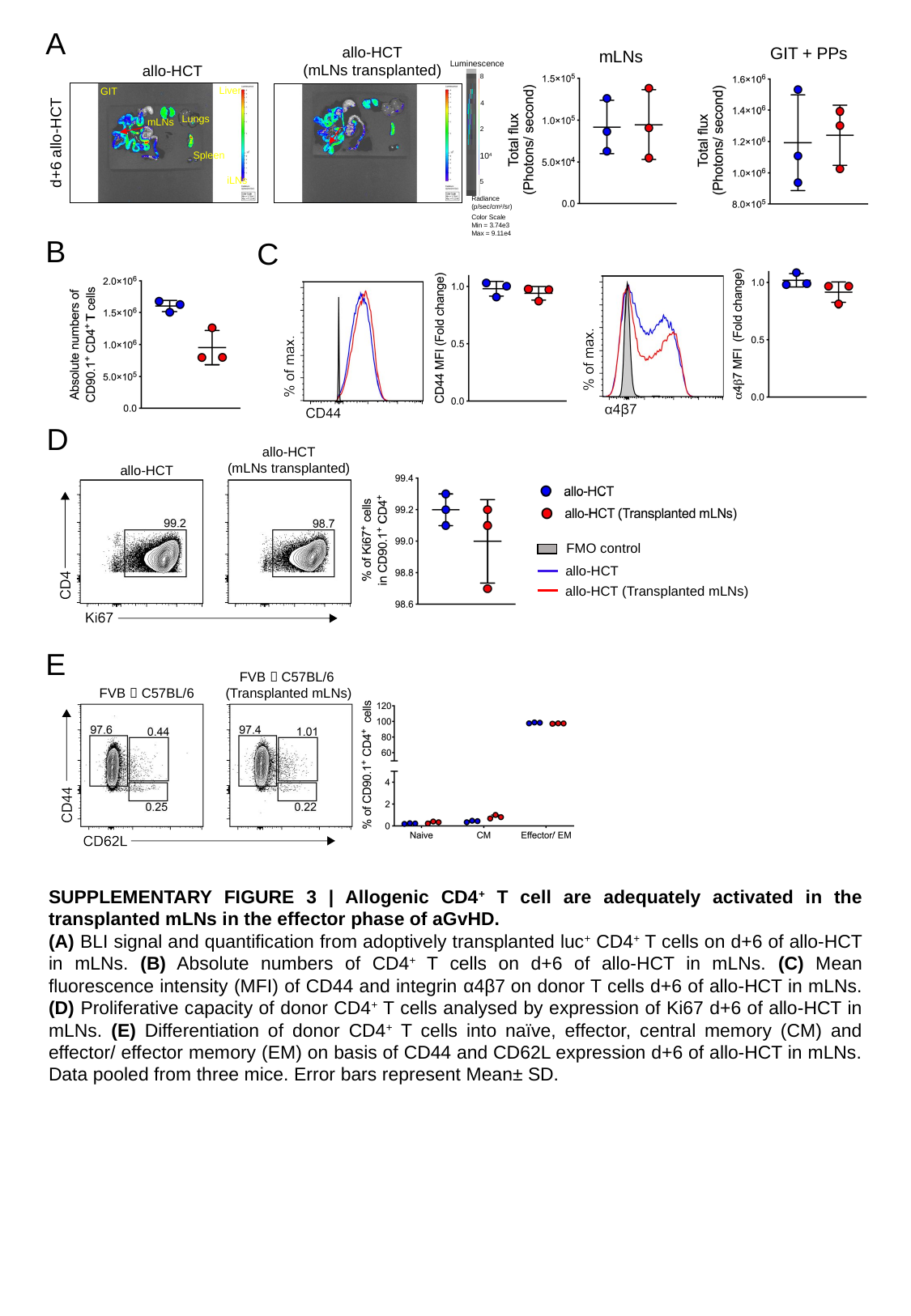

A
allo-HCT
(mLNs transplanted)
GIT + PPs
mLNs
Luminescence
allo-HCT
8
Liver
GIT
4
Lungs
mLNs
2
d+6 allo-HCT
Spleen
104
iLNs
5
Radiance
(p/sec/cm2/sr)
Color Scale
Min = 3.74e3
Max = 9.11e4
B
C
D
allo-HCT
(mLNs transplanted)
allo-HCT
FMO control
allo-HCT
allo-HCT (Transplanted mLNs)
E
FVB  C57BL/6
(Transplanted mLNs)
FVB  C57BL/6
SUPPLEMENTARY FIGURE 3 | Allogenic CD4+ T cell are adequately activated in the transplanted mLNs in the effector phase of aGvHD.
(A) BLI signal and quantification from adoptively transplanted luc+ CD4+ T cells on d+6 of allo-HCT in mLNs. (B) Absolute numbers of CD4+ T cells on d+6 of allo-HCT in mLNs. (C) Mean fluorescence intensity (MFI) of CD44 and integrin α4β7 on donor T cells d+6 of allo-HCT in mLNs. (D) Proliferative capacity of donor CD4+ T cells analysed by expression of Ki67 d+6 of allo-HCT in mLNs. (E) Differentiation of donor CD4+ T cells into naïve, effector, central memory (CM) and effector/ effector memory (EM) on basis of CD44 and CD62L expression d+6 of allo-HCT in mLNs. Data pooled from three mice. Error bars represent Mean± SD.

## Slide 4
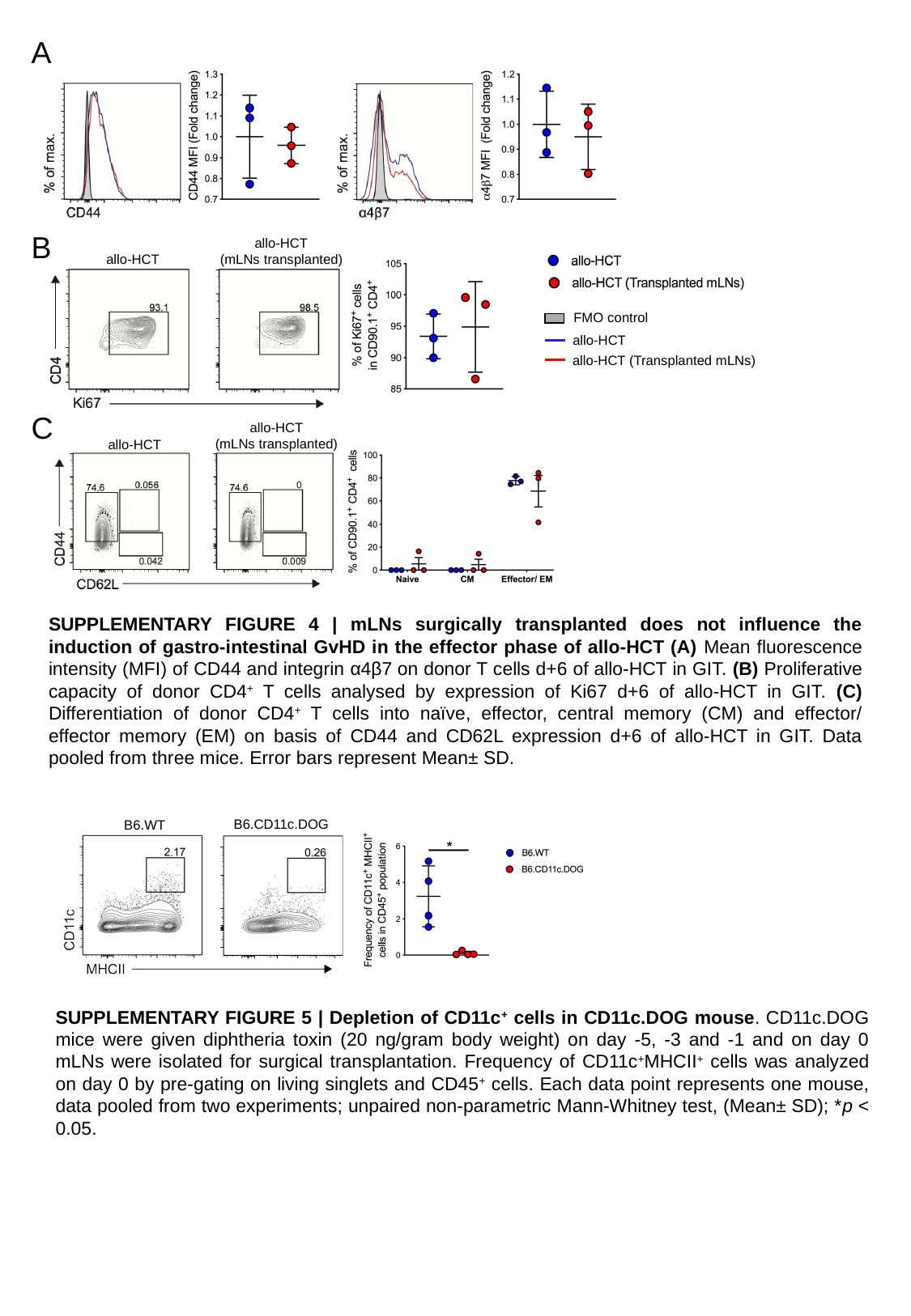

A
B
allo-HCT
(mLNs transplanted)
allo-HCT
FMO control
allo-HCT
allo-HCT (Transplanted mLNs)
C
allo-HCT
(mLNs transplanted)
allo-HCT
SUPPLEMENTARY FIGURE 4 | mLNs surgically transplanted does not influence the induction of gastro-intestinal GvHD in the effector phase of allo-HCT (A) Mean fluorescence intensity (MFI) of CD44 and integrin α4β7 on donor T cells d+6 of allo-HCT in GIT. (B) Proliferative capacity of donor CD4+ T cells analysed by expression of Ki67 d+6 of allo-HCT in GIT. (C) Differentiation of donor CD4+ T cells into naïve, effector, central memory (CM) and effector/ effector memory (EM) on basis of CD44 and CD62L expression d+6 of allo-HCT in GIT. Data pooled from three mice. Error bars represent Mean± SD.
B6.CD11c.DOG
B6.WT
SUPPLEMENTARY FIGURE 5 | Depletion of CD11c+ cells in CD11c.DOG mouse. CD11c.DOG mice were given diphtheria toxin (20 ng/gram body weight) on day -5, -3 and -1 and on day 0 mLNs were isolated for surgical transplantation. Frequency of CD11c+MHCII+ cells was analyzed on day 0 by pre-gating on living singlets and CD45+ cells. Each data point represents one mouse, data pooled from two experiments; unpaired non-parametric Mann-Whitney test, (Mean± SD); *p < 0.05.
